# Telemedical stroke care significantly improves patient outcome in rural areas: Long-term analysis of the German NEVAS network

**DOI:** 10.1177/17474930241234259

**Published:** 2024-02-28

**Authors:** Ilias Masouris, Lars Kellert, Cauchy Pradhan, Johannes Wischmann, Roman Schniepp, Robert Müller, Leonard Fuhry, Gerhard F Hamann, Thomas Pfefferkorn, Jan M Rémi, Florian Schöberl

**Affiliations:** 1 Department of Neurology, LMU University Hospital, LMU Munich, Munich, Germany; 2 Department of Neurology and Neurological Rehabilitation, Bezirkskrankenhaus Guenzburg, Günzburg, Germany; 3 Department of Neurology, Klinikum Ingolstadt, Ingolstadt, Germany

**Keywords:** Telemedicine network, stroke, mRS, intravenous thrombolysis

## Abstract

**Background:**

Comprehensive stroke centers (CSC) offer state-of-the-art stroke care in metropolitan centers. However, in rural areas, sufficient stroke expertise is much scarcer. Recently, telemedical stroke networks have offered instant consultation by stroke experts, enabling immediate administration of intravenous thrombolysis (IVT) on-site and decision on thrombectomy. While these immediate decisions are made during the consult, the impact of the network structures on stroke care in spoke hospitals is still not well described.

**Aims:**

This study was performed to determine if on-site performance in rural hospitals and patient outcome improve over time through participation and regular medical staff training within a telemedical stroke network.

**Methods:**

In this retrospective study, we analyzed data from stroke patients treated in four regional hospitals within the telemedical Neurovascular Network of Southwest Bavaria (NEVAS) between 2014 and 2019. We only included those patients that were treated in the regional hospitals until discharge at home or to neurorehabilitation. Functional outcome (modified Rankin scale) at discharge, mortality rate and periprocedural intracranial hemorrhage served as primary outcome parameters. Door-to-imaging and door-to-needle times were secondary outcome parameters.

**Results:**

In 2014–2019, 5,379 patients were treated for acute stroke with 477 receiving IVT. Most baseline characteristics were comparable over time. For all stroke patients, door-to-imaging times increased over the years, but significantly improved for potential IVT candidates and those finally treated with IVT. The percentage of patients with door-to-needle time <30 min increased from 10% to 25%. Clinical outcome at discharge improved for all stroke patients treated in the regional hospitals. Particularly for patients treated with IVT, good clinical outcome (modified Rankin scale 0–2) at discharge increased from 2014 to 2019 by 19% and mortality rates dropped from 13% to 5%.

**Conclusions:**

24-h/7-day telemedical support and regular on-site medical staff training within a structured telemedicine stroke network such as NEVAS significantly improve on-site stroke care in rural areas, leading to a considerable benefit in clinical outcome.

**Data access statement:**

The data that support the findings of this study are available upon reasonable request and in compliance with the local and international ethical guidelines.

## Introduction

Timely acute stroke management, including intravenous thrombolysis (IVT), improves functional outcome considerably.^1,2^ In metropolitan areas, comprehensive stroke centers (CSC) offer state-of-the-art stroke care. However, in rural areas with small to medium regional hospitals, sufficient stroke expertise is much scarcer, resulting in either longer transport times to the next CSC or less routine of acute stroke management on-site. In recent years, established telemedical stroke networks offered 24 h/7 days stroke expertise to regional hospitals in rural areas, enabling immediate administration of IVT on-site.^3,4^ Previous studies suggested a benefit for stroke patients in rural areas by telemedical stroke concepts.^5–7^ However, valid data on the effect of long-term participation in a telemedicine stroke network on on-site performance and clinical outcome are largely missing so far.

The NEuroVAScular Network of Southwest Bavaria (NEVAS) is a structured telemedicine stroke network, where patients with acute stroke are telemedically presented to stroke experts with their clinical symptoms and CT-scans per videostream. In previous studies, we could show that patients eligible for mechanical thrombectomy benefit from the “drip-and-ship” concept within our network.^8,9^ However, with around 90%, the majority of stroke patients remain in the regional hospitals receiving stroke care on-site, for example, because MT is not indicated. Therefore, CSCs regularly train the medical staff in the regional hospitals of our network to provide the best standard of stroke care.

## Aims/hypothesis

In this study, we systematically analyzed data of stroke patients treated in four rural hospitals of NEVAS from 2014 to 2019. Baseline characteristics, key workflow time intervals as well as critical outcome-related data such as the modified ranking scale (mRS) at discharge, mortality rates and intracranial hemorrhage rates were compared over the years to examine if on-site performance and clinical outcomes had improved over time through participation in NEVAS.

## Methods

### Supraregional telemedicine stroke network NEVAS

The NEVAS stroke telemedicine network was founded in 2014 in Bavaria, Germany, providing specialized neurovascular care for ca. 2,9000,000 people in southwest Bavaria. The network consists of 3 CSCs (University Hospital, Ludwig-Maximilians-University Munich and the non-universitary maximum care hospitals, Klinikum Ingolstadt and district hospital Guenzburg). Each CSC provides telemedical stroke support to non-CSC hospitals in local proximity. The attributed hospitals are either level I hospitals (i.e. hospitals providing basic care without neurological departments) or level II hospitals (i.e. hospitals with neurological departments but not covering the full spectrum of specialized neurovascular care, particularly no mechanical thrombectomy). A detailed map of the network is provided in Feil et al.^9^ The three CSCs consult the regional hospitals on a 24 h-basis telemedically for acute stroke patients. Patients with suspected stroke are presented immediately via videostream—together with their stroke CT-imaging—and clinical advice is given by the CSCs. All patients not eligible for mechanical thrombectomy, revascularization therapy or needing transportation to the CSCs for other reasons remain in the regional hospitals until discharge.

For continued stroke teaching, theoretical and practical training is performed 4 to 5 times per year on-site as well as two times per year at the Hospital of the Ludwig–Maximilians–University Munich by the stroke teams of the CSCs for the whole medical staff involved in stroke treatment (physicians, nurses, physical, behavioral, speech therapists) to continuously improve clinical care on-site. All hospitals within the network are trained with the same frequency, workload and contents by their assigned CSC in a standardized manner. In addition, performance parameters are monitored and critically discussed in structured assessments for possible improvements.

### Study design

We performed a retrospective multi-center cross-sectional study at the Department of Neurology of the University Hospital, Ludwig-Maximilians-University Munich.

### Patient data

We included all consecutive patients (age > 18 years) with acute stroke that were treated between 2014 and 2019 in four regional hospitals within the NEVAS network, that are assigned for telemedical consultation to the University Hospital, Ludwig–Maximilians–University Munich. Patients secondarily transported to our CSC were excluded as the focus of this study is the on-site stroke care quality. We divided this overall period in three time intervals (2014-2015, 2016-2017 and 2018-2019), which we compared for various parameters. We retrospectively analyzed comprehensive stroke treatment quality data that is routinely collected by partner hospitals according to national mandatory quality assurance guidelines. It encompasses biometrical data (age, sex); cardiovascular risk factors, diagnosis, neurological examination, imaging, essential time intervals. Stroke severity was assessed by the National Institutes of Health Stroke Scale (NIHSS) and functional outcome by the modified Rankin Scale (mRS). mRS at discharge, mortality rate and periprocedural intracranial hemorrhage served as primary outcome parameters. Door-to-imaging, door-to-needle times were secondary outcome parameters. Regarding functional outcome, the overall mRS shift over the years was analyzed; furthermore, excellent outcome was defined as mRS 0-1, good outcome as mRS 0–2. The outcome parameters were compared over time for all stroke patients and secondarily for those undergone IVT.

### Imaging

Standard imaging of patients with acute stroke consisted of an initial CT imaging including non-contrast CT and CT angiography. CT-perfusion was not performed in any of the included cases, as it was not provided routinely in our spoke hospitals in the analyzed time period. Within 24 h after IVT, each patient underwent CT or magnetic resonance imaging (MRI) to rate the extent of an ischemic lesion and to detect intracranial hemorrhage.

### Indications for IVT treatment

IVT eligibility was assessed during the telemedical consultation according to the national and international IVT guidelines taking account the symptom time window, CT scan and contraindications.

### Statistics

Statistical tests were performed using the Prism Software (GraphPad) and SPSS (IBM Corp, Armonk, NY). P-values < 0.05 were considered significant. Each variable is displayed with median and interquartile range (IQR). Univariate analysis was conducted for comparison between three groups, using the Kruskal–Wallis and subsequently Dunn’s multiple comparison test as well as the χ²-test, where appropriate. To determine association with outcome and safety parameters, ordinal (shift analysis) and binary logistic regression models were used. All regression analysis models were adjusted for age, sex, NIHSS, symptom onset to admission time, and door-to-needle time as well as all variables that were significantly different between groups in univariate analysis which were diabetes mellitus, atrial fibrillation, hypertension, and days in hospital. Effect estimates are displayed as adjusted odds ratios (OR) with 95% confidence intervals (CI). Propensity score matching was carried out for stroke patients treated with IVT within NEVAS and an equivalent patient cohort treated directly in our CSC, using logistic regression analysis based on age, sex, and NIHSS at baseline to estimate the propensity score with a caliber of 0.2. Nearest neighbor matching was used for the time periods 2014–2015 and 2018–2019. Age, sex, and NIHSS were well balanced between both groups in both time periods.

## Results

### Total stroke patients

5379 patients were treated for acute stroke in the four regional hospitals between 2014 and 2019 (Table 1). In comparison to the first 2 years, the total number of stroke patients increased from ca. 770 to 970 per year (ca. 25). Most baseline parameters were similar in all time periods. The NIHSS at onset was slightly higher in 2014–2015 (median 3 vs 2 and 2, p = 0.0001). Symptom onset to admission time was comparable at a median of 3–4 h, while in ca. 10% of the patients, symptom onset was unknown. Hypertension, diabetes and atrial fibrillation prevalence rates were higher in 2014–2015 compared to the other time periods.

**Table 1.** Baseline patient characteristics for stroke patients that were treated in rural hospitals within the telemedicine network NEVAS over the years.

Baseline characteristics	2014–2015, n = 1544	2016–2017, n = 1950	2018–2019, n = 1885	p-value
TIA/Stroke	615/929	821/1129	663/1222	
Age, years, median (IQR)	77 (69–85)	77 (66–83)	78 (67–84)	**0.0006**
Sex, female, n (%)	784 (50.7%)	953 (48.9%)	891 (47.3%)	0.120
Premorbid condition^a^, median (IQR)	1 (1–1)	1 (1–1)	1 (1–1)	0.160
NIHSS at onset, median (IQR)	3 (1–9)	2 (1–6)	2 (0–5)	**0.0001**
Risk factors, n (%)			*n* *=* *1871*	–
Hypertension	1161 (75.2%)	1313 (67.3%)	1242 (66.4%)	**0.0001**
Diabetes mellitus	381 (24.7%)	405 (20.7%)	382 (20.4%)	**0.004**
Atrial fibrillation	424 (27.5%)	461 (23.6%)	443 (23.7%)	**0.028**
CT %	100%	100%	100%	–
CT angiography n (%)	100%	100%	100%	–
Days in hospital, median (IQR)	6 (4–10)	6 (4–9)	6 (4–9)	**0.0001**
Symptom onset unknown, %	12.2%	8%	10.3%	–
Symptom onset to admission time^b^	4 (2–7)	4 (2–7)	4 (2–7)	0.07
IVT administration %	8.9%	7.9%	9.8%	–

TIA: transitory ischemic attack; IQR: interquartile range; CT: computed tomography.

Biometrical and clinical data of the included stroke patients were compared between time periods. Univariate analysis was performed using the Mann–Whitney-U-test and the Chi²-test, where appropriate. Significant p-values < 0.05 are in bold letters.

a Premorbid condition defined as (1) independent at home, (2) nursing at home (3) living in nursing home.

b Symptom onset to admission time defined as 1 = ⩽ 1 h, 2 = > 1–2 h, 3 = > 2–3 h, 4 = > 3–4 h, 5 = > 4–5 h, 6 = > 5–6 h, 7 = > 6–24 h, 8 = > 24–48 h, 9 = > 48 h.

#### Clinical outcome, complications, and key workflow time intervals in acute stroke management

**Figure 1.**
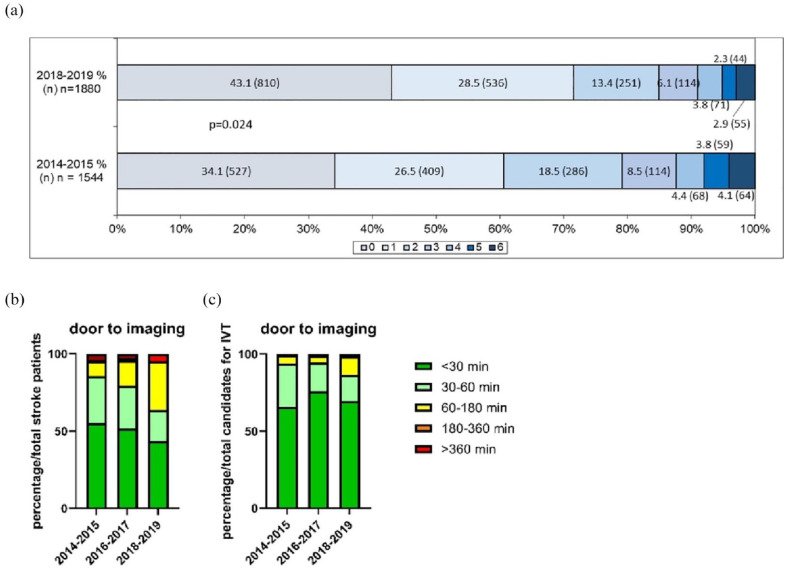
Key workflow time intervals and clinical outcome for stroke patients within the NEVAS telemedicine stroke network. (a) Shift analysis of mRS score at discharge for all stroke patients treated within the NEVAS network in 2014–2015 versus 2018–2019. The absolute number and the percentage to the total number of each patient group are displayed for each mRS Score. (b, c) Door-to-imaging time distribution among all stroke patients (a) and those eligible for IVT (b). Door-to-imaging times are displayed as time ranges: (<30 min/dark green, 30–60 min/light green, 60–180 min/yellow, 180–360 min/orange, >360 min red). The percentage of stroke patients that received a CT scan within each time range is depicted.

Clinical outcome at discharge was assessed for all patients by the mRS score (Table 2). The mRS was available for 1544 patients (100%) in 2014–2015, 1948 (99.9%) in 2016–2017, and 1880 (99.7%) in 2018–2019. In the univariate analysis, the primary outcome (mRS 0-2) increased over the years (79.1% vs 82.3% and 84.9%, p = 0.0001). The percentage of patients with mRS 0–1 similarly increased over time. The mRS shift analysis demonstrated a significant shift toward better functional outcomes in 2018–2019 (OR 1.19; 95% CI 1.02–1.39; p = 0.024; Figure 1(a)). Dichotomized regression analysis for the years 2014–2015 and 2018–2019 also revealed that the odds of achieving mRS 0–1 (OR 1.24; 95% CI 1.01–1.53; p = 0.045) and mRS 0–2 (OR 1.79; 95% CI 1.37–2.33; p = 0.001) at discharge were significantly higher in 2018-2019. Mortality at discharge gradually decreased (from 4.1% to 3.4% and 2.9%) without reaching significance (p = 0.151), the adjusted OR was 1.80 (95% CI 1.08–3.01; p = 0.025) for 2014–2015. All regression analysis models were adjusted for age, sex, NIHSS, symptom onset to admission time, and door-to-needle time as well as diabetes mellitus, atrial fibrillation, hypertension, and days in hospital.

**Table 2.** Clinical follow-up of all stroke patients using the modified Rankin scale (mRS).

mRS at discharge, n (%)					
	2014–2015 (n = 1544, 100%)	2016–2017 (n = 1948, 99.9%)	2018–2019 (n = 1880, 99.7%)	OR (95% CI) 14–15 vs 18–19	p value
Univariate analysis					
mRS 0–1	936 (60.6%)	1344 (69%)	1345 (71.5%)		**0.0001**
mRS 0–2	1222 (79.1%)	1603 (82.3%)	1596 (84.9%)		**0.0001**
Mortality (mRS 6)	64 (4.1%)	67 (3.4%)	55 (2.9%)		0.151
Regression analysis					
mRS 0–6 shift analysis				1.19 (1.02–1.39)	**0.024**
mRS 0–1				1.24 (1.01–1.53)	**0.045**
mRS 0–2				1.79 (1.37–2.33)	**0.001**
Mortality (mRS 6)				1.80 (1.08–3.01)	**0.025**

OR: odds ratio; CI: confidence interval.

Stroke patient clinical outcome at discharge was assessed using the mRS score. The percentage of patients with mRS 0–1, 0–2 as well mortality rates were compared between the years 2014–2015 and 2018–2019. Univariate analysis was performed using the Chi²-test. Ordinal regression analysis and binary logistic regression analysis (dichotomized mRS) was adjusted for age, sex, NIHSS, diabetes mellitus, atrial fibrillation, hypertension, symptom onset to admission time, days in hospital and door-to-needle time. Significant p-values < 0.05 are in bold letters.

Regarding workflow, we compared the door-to-imaging times over the years for all acute stroke patients. The exact time intervals were not available, instead they were coded as follows: <30, 30–60, 60–180, 180–360, and > 360 min. The percentage of patients receiving a CT scan in <30 and <60 min after admission declined from 55.2% and 85.7% in 2014–2015% to 43.4% and 63.7% in 2018–2019 respectively (Figure 1(b)). We further analyzed only those stroke patients that were potentially eligible for IVT, i. e. a symptom–time window <4 h and an NIHSS above 0. The percentage of patients receiving a CT scan in <30 and <60 min after admission increased from 2014–2015 to 2016–2017 (from 65.6% to 75.8%; p = 0.0001, and 93.7% to 94.7%; p = 0.118, respectively; Figure 1(c)), however showed a decline in 2018–2019 (69.5%; p = 0.009% and 86.6%; p = 0,001).

### Stroke patients treated with IVT

A total of 477 patients (8.9%) received IVT between 2014 and 2019 (Table 3). IVT indication by telemedical consultation decreased from 86.2% in 2014–2015 to 72.3% in 2018–2019 due to the hiring of local neurologists in the regional hospitals for the regular shift hours. Most baseline characteristics were similar in all time periods. NIHSS at onset was slightly, albeit not significantly, higher in 2014–2015 (2014–2015: median 9 vs 2016–2017: median: 7 and 2018–2019: median 8, p = 0.06). Median symptom onset to admission time was similar on all groups at 1–2 h with a significant distribution toward a higher range in 2016–2017 and 2018–2019 (p = 0.0001). Hypertension rates were higher in 2014–2015 compared to the other time periods (p = 0.0001).

**Table 3.** Baseline patient characteristics for stroke patients that were administered intravenous thrombolysis (IVT) in the telemedicine network NEVAS over the years.

Baseline characteristics for IVT patients	2014–2015 n = 138	2016–2017 n = 155	2018–2019 n = 184	p-value
Age, years, median (IQR)	79 (67–87)	79 (68–85)	77 (69–83)	0.681
Sex, female, n (%)	69 (50%)	74 (47.7%)	81 (44%)	0.552
Premorbid condition^a^, median (IQR)	1 (1–1)	1 (1–1)	1 (1–1)	0.600
NIHSS at onset, median (IQR)	9 (4–18)	7 (4–12)	8 (4–12)	0.064
Risk factors, n (%)			n = 177	–
Hypertension	106 (76.8%)	91 (58.7%)	93 (52.5%)	**0.0001**
Diabetes mellitus	31 (22.4%)	30 (19.7%)	30 (16.9%)	0.470
Atrial fibrillation	46 (33.3%)	41 (26.4%)	39 (22%)	0.080
CT %	100%	100%	100%	–
CT angiography n (%)	100%	100%	100%	–
Days in hospital, median (IQR)	7 (5–11)	7 (4–11)	7 (3.5–11)	0.822
IVT decision through telemedical consultation, %	86.2%	73.1%	72.3%	–
Symptom onset to admission time^b^	2 (1–3)	2 (2–2)	2 (2–3)	**0.0001**
ICH, n (%)	3 (2.2%)	4 (2.6%)	6 (3.3%)	0.831

IQR: interquartile range; CT: computed tomography; ICH: intracranial hemorrhage.

Biometrical and clinical data of the included stroke patients were compared between time periods. Univariate analysis was performed using the Mann–Whitney U test and the χ²-test, where appropriate. Significant p-values < 0.05 are in bold letters.

a Premorbid condition defined as (1) independent at home, (2) nursing at home (3) living in nursing home.

b Symptom onset to admission time defined as 1 ⩽ 1 h, 2 ⩾ 1–2 h, 3 ⩾ 2–3 h, 4 ⩾ 3–4 h.

#### Clinical outcome, complications and key workflow time intervals in acute stroke management

**Figure 2.**
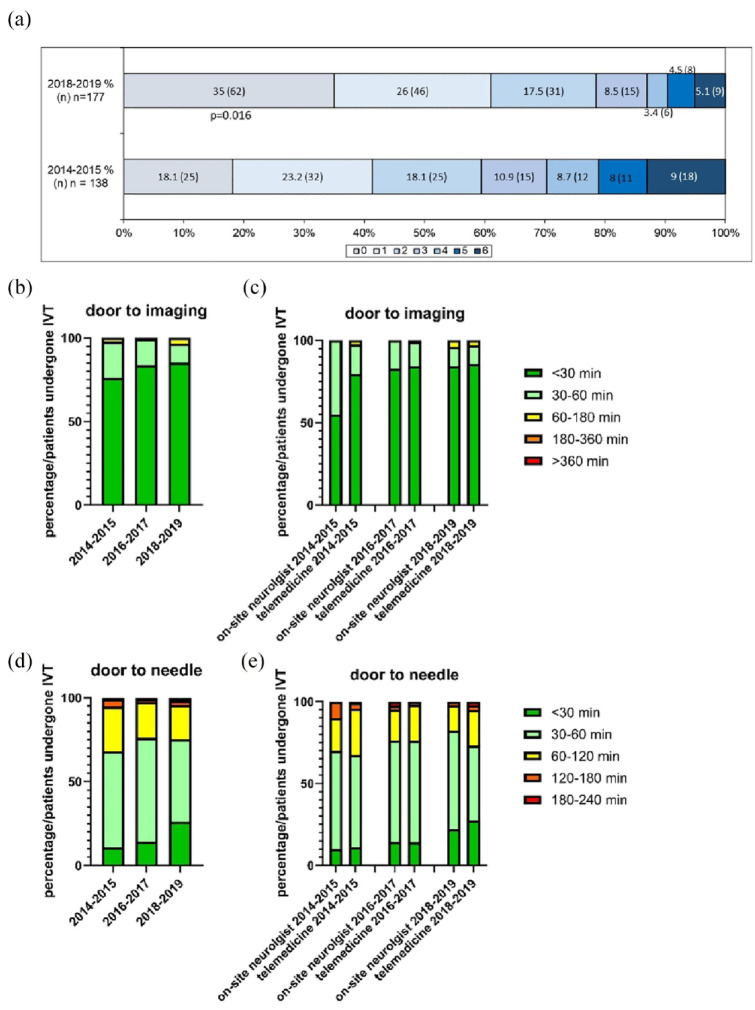
Key workflow time intervals and clinical outcome for stroke patients undergone intravenous thrombolysis (IVT) treatment within the NEVAS telemedicine stroke network. (a) Shift analysis of mRS score at discharge for stroke patients treated with IVT within the NEVAS network in 2014–2015 versus 2018–2019. The absolute number and the percentage to the total number of each patient group are displayed for each mRS Score. (b–e) Door-to-imaging (b, c) and door-to-needle (d, e) time distribution among all stroke patients treated with IVT (b, d), and comparing IVT indication by on-site neurologist versus telemedical consultation (c, e). Door-to-imaging times are displayed as time ranges: (<30 min/dark green, 30–60 min/light green, 60–180 min/yellow, 180–360 min/orange, >360 min red). The percentage of stroke patients that received a CT scan within each time range is depicted.

The mRS was available for 138 patients (100%) in 2014–2015, 155 (100%) in 2016–2017 and 177 (96.2%) in 2018–2019 (Table 4). Good clinical outcome significantly increased over time (mRS 0–2: 78.5% vs 72.3% and 59.4%, p = 0.0002) The percentage of patients with mRS 0–1 similarly increased over time. The mRS shift analysis demonstrated a significant shift toward better functional outcomes in 2018–2019 (OR 1.72; 95% CI 1.11–2.64; p = 0.016; Figure 2(a)). Dichotomized regression analysis between 2014–2015 and 2018–2019 also revealed that the odds of achieving mRS 0–1 (OR 1.82; 95% CI 1.04–3.19; p = 0.035) and mRS 0–2 (OR 1.95; 95% CI 1.07–3.56; p = 0.030) at discharge were significantly higher in 2018–2019. Mortality rates significantly decreased (from 13% in 2014–2015 to ca. 5% in the following years, p = 0.012) with an OR of 1.86 for 2014–2015, which was, however, not significant (p = 0.277). All regression analysis models were adjusted for age, sex, NIHSS, symptom onset to admission time and door-to-needle time as well as hypertension. mRS 0–2 and mortality rates improved both for consultations by our network and by on-site neurologists over the years. For consultations through our network, the mRS 0–2 rate improved from 59.3% in 2014–2015 to 81.3% in 2018–2019 and the mortality rate sank from 13.6% to 6.3%. For consultations by on-site neurologists, the mRS 0–2 rate improved from 60% to 71.3% and the mortality rate sank from 10% to 2% in the same time period (data not shown).

**Table 4.** Clinical follow-up of stroke patients undergone IVT using the modified Rankin scale (mRS).

mRS at discharge, n (%)					
	2014–2015 (n = 138, 100%)	2016–2017 (n = 155, 100%)	2018–2019 (n = 177, 96.2%)	OR (95% CI) 14–15 vs 18–19	p value
Univariate analysis					
mRS 0–1	57 (41.3%)	88 (56.8%)	108 (61%)		**0.0005**
mRS 0–2	82 (59.4%)	112 (72.3%)	139 (78.5%)		**0.0002**
Mortality (mRS 6)	18 (13%)	8 (5.2%)	9 (5.1%)		**0.012**
Regression analysis					
mRS 0–6 shift analysis				1.72 (1.11–2.64)	**0.016**
mRS 0–1				1.82 (1.04–3.19)	**0.035**
mRS 0–2				1.95 (1.07–3.56)	**0.030**
Mortality (mRS 6)				1.86 (0.61–5.69)	0.277

OR: odds ratio; CI: confidence interval.

Stroke patient clinical outcome at discharge was assessed using the mRS score. The percentage of patients with mRS 0–1, 0–2 as well mortality rates were compared between the years 2014–2015 and 2018–2019. Univariate analysis was performed using the χ²-test. Ordinal regression analysis and binary logistic regression analysis (dichotomized mRS) was adjusted for age, sex, NIHSS, hypertension, symptom onset to admission time and door-to-needle time. Significant p-values < 0.05 are in bold letters.

Door-to-imaging times for stroke patients that underwent IVT improved over the years (Figure 2(b)). Particularly, the percentage of patients receiving a CT scan in <30 min after admission increased from 76.1% in 2014–2015 to 83.9% in 2016–2017 and 85.3% in 2018–2019 (p = 0.103 and 0.035, respectively). Comparing door-to-imaging times in IVT cases indicated by the on-site neurologist and those indicated by telemedical consultation, both improved over the years (55% to 84% and 80% to 85% in <30 min, respectively; Figure 2(c)). The percentage of patients with door-to-needle times <30 and <60 min also increased from 2014–2015 to 2018–2019 (from 10.9% and 68.1% to 26.2% and 75.4%, respectively; Figure 2(d)). The improvement was observed both in cases consulted by the on-site neurologist and by telemedical consultation (10% to 29% and 11% to 26% in <30 min, 70% to 86% and 67% to 80% in <60 min, respectively; Figure 2(e)). Concerning post-IVT complications, intracranial hemorrhage rates were low and comparable over the years at ca. 2–3% (Table 3).

#### Propsensity score matched analysis

We compared our IVT-treated NEVAS cohort with a matched cohort treated directly in our CSC in the years 2014–2015 and 2018–2019 using propensity score matching (Supplemental Table 1). In the new matched groups, the percentage of NEVAS patients with mRS 0–2 was lower in 2014–2015 and higher in 2018–2019 than of the CSC patients, however without reaching significance (p = 0.553 and 0.251, respectively). The percentage of NEVAS patients with mRS 0–2 improved from 63.2% to 75.8% between 2014–2015 and 2018–2019 with significantly higher odds in 2018–2019 (OR 2.15; 95% CI 1.20–3.85; p = 0.020), while for the CSC patients the percentage improved by a smaller amount (4.8%, 68% to 72.8%) in the same time period, without reaching significance (OR 1.15; 95% CI 0.50–2.65; p = 0.747).

## Discussion

In this study, we evaluated data from patients with acute stroke treated within the NEVAS telemedicine network from 2014 to 2019 to examine if stroke management and clinical outcome improved over the years. Our key findings were the following: (a) clinical outcome at discharge improved for all stroke patients over the years despite an increase in the door-to-imaging time; (b) door-to-imaging times were significantly shorter for potential IVT candidates and those treated with IVT over the years; (c) door-to-needle times significantly improved over the years; (d) the percentage of patients treated with IVT with a good clinical outcome at discharge increased by 20% from 2014 to 2019 and mortality rates at discharge decreased from 13.0% to 5.1%.

Good clinical outcome at discharge, defined as mRS 0–2, improved considerably over the years, particularly for IVT-treated patients. With ca. 78% of patients reaching a good clinical outcome at discharge after IVT treatment in 2018–2019, the findings are in the upper field of good outcome rates of other major IVT studies. However, outcome data are usually analyzed at 3 months follow-up and thus are not entirely comparable with our results.^10–12^ Beside shorter door-to-imaging and door-to-needle time intervals observed here, there were other factors possibly contributing to this improvement. The CSCs within our telemedicine network are not only tasked to provide instant telemedical consultation, but also to train medical staff in the regional hospitals regularly to improve acute and stationary stroke management on-site and stepwise establish regional stroke units with excellent stroke care. As deficit-targeted care and early physiotherapy, ergotherapy with mobilization and speech therapy are important factors for improving clinical outcome after stroke,^13^ important parts of the network training focuses on these sectors. This training is therefore a contributing factor to the improvement of clinical outcome at discharge. In addition, due to a retrospective data analysis, there were few differences in baseline parameters that may have influenced the clinical outcome. Further premorbid mRS was not available for our cohort. However, all factors were considered in the adjusted regression analysis. Another possible contributing factor could be a global improvement in stroke care over the years. To address this issue, we compared our results with a propensity matched patient cohort of our CSC and found between 2014-2015 and 2018-2019 an improvement of 15.6% in the NEVAS patients compared to 4.8% in the CSC patients. Furthermore, two other studies conducted in the same time period also showed an improvement of 2–3% in clinical outcome of IVT-treated stroke patients.^14,15^ Thus, the gradual improvement in good clinical outcome can mostly be contributed to the NEVAS network.

Since its establishment in 2014, the NEVAS network noticed an increase in the number of stroke patients treated acutely through telemedical consultation of around 25%. This is probably reflected in the observed overall increase of the door-to-imaging time for all stroke patients over the years. Beside the increased influx of stroke patients over the years, another contributing factor is possibly the globally observed over-crowding of the emergency departments in the last years, resulting in the need of better triaging of medical emergencies.^16^ Indeed, when concentrating on stroke patients admitting within a time window of 4.5 h with ongoing neurological symptoms and thus eligible for IVT, door-to-imaging times were shorter and improved from 2014–2015 to 2016–2017 considerably. These results indicate that physicians in the regional hospitals have learnt through regular training by the NEVAS network to prioritize stroke patients eligible for acute treatment despite an increasing workload in the emergency department.

With an average IVT rate of 8–9% among all stroke patients, our results are in line with IVT rates from other studies.^17^ For patients that were administered IVT, both door-to-imaging and door-to-needle times significantly improved over the years. Notably, both time intervals were comparable for cases consulted by the on-site neurologist and those per telemedical consultation. Thus, telemedically assisted acute stroke management has not an inferior workflow tempo than on-site consultation and improves through training and experience within the network. Regarding safety, intracranial hemorrhage (ICH) rates remained low over the years and were with 2–3% lower than in major IVT trials,^2,10,18^ indicating that IVT administration under telemedical guidance is safe. Furthermore, the mortality rate for all stroke patients dropped from 4.1% to 2.9% from 2014–2015 to 2018–2019 and even more substantially for those receiving IVT, as the rate dropped from 13% to 5.1% and was somewhat lower than those reported in major IVT trials.^2,10,18^ Thus, acute stroke treatment including IVT via a telemedical stroke network is both effective and safe with continuous improvement over time.

In conclusion, stroke patients treated in regional hospitals greatly benefit from a structured telemedicine stroke network such as NEVAS through 24 h/7d telemedical support coverage. Regular on-site training of medical staff leads to improved performance on stroke acute management and care over time.

## Supplemental Material

sj-docx-1-wso-10.1177_17474930241234259 – Supplemental material for Telemedical stroke care significantly improves patient outcome in rural areas: Long-term analysis of the German NEVAS network Supplemental material, sj-docx-1-wso-10.1177_17474930241234259 for Telemedical stroke care significantly improves patient outcome in rural areas: Long-term analysis of the German NEVAS network by Ilias Masouris, Lars Kellert, Cauchy Pradhan, Johannes Wischmann, Roman Schniepp, Robert Müller, Leonard Fuhry, Gerhard F Hamann, Thomas Pfefferkorn, Jan M Rémi and Florian Schöberl in International Journal of Stroke
