# Supplementary material for: Telemedical stroke care significantly improves patient outcome in rural areas: Long-term analysis of the German NEVAS network
Source: Int J Stroke. 2024 Feb 28;19(5):577–86. doi: 10.1177/17474930241234259 (PMC11134988; doi:10.1177/17474930241234259)
Supplement: sj-docx-1-wso-10.1177_17474930241234259 – Supplemental material for Telemedical stroke care significantly improves patient outcome in rural areas: Long-term analysis of the German NEVAS network [file sj-docx-1-wso-10.1177_17474930241234259.docx]

**Supplemental Table 1. Propensity score matching for stroke patients treated with IVT.** Stroke patients treated with IVT within the NEVAS network were matched with an equivalent patient cohort treated with IVT in our comprehensive stroke center (CSC) using propensity analysis. Propensity score matching was carried out using logistic regression analysis based on age, sex and NIHSS at baseline to estimate the propensity score with a caliber of 0.2. Nearest neighbor matching was used, matching IVT-treated patients of NEVAS and our CSC for the time periods 2014-2015 and 2018-2019. Clinical outcome at discharge was assessed using the modified Ranikn Scale (mRS). Univariate analysis was performed using the Chi²-test. Binary logistic regression analysis was adjusted for age, sex, NIHSS, arterial hypertension, symptom onset to admission time and door-to-needle time. Significant p-values <0.05 are in bold letters. OR = Odd´s ratio; CI = Confidence interval

| **mRS 0-2**  **n/total (%)** | **NEVAS patients** | **CSC patients** | **p-value**  **Chi^2^-test** |
| --- | --- | --- | --- |
| 2014-2015 | 79/125 (63.2%) | 30/44 (68%) | 0.553 |
| 2018-2019 | 138/182 (75.8%) | 78/107 (72.9%) | 0.251 |
| Binary logistic regression |  |  |  |
| Odds ratio (95% CI) | 2.15 (1.20 – 3.85) | 1.15 (0.50 – 2.65) |  |
| p-value | **0.020** | 0.747 |  |
